# 2174: In silico prediction of NS1 structure and influenza A virus pathogenesis

**DOI:** 10.1017/cts.2017.26

**Published:** 2018-05-10

**Authors:** Joshua Klonoski, Julio C. Facelli

## Abstract

OBJECTIVES/SPECIFIC AIMS: This poster presents preliminary results of using in silico approaches to predict a priori, based on sequence alone, the pathogenesis of novel influenza A virus. METHODS/STUDY POPULATION: Here we analyzed the structure of the NS1 protein of 11 strains of well characterized influenza A virus with known pathogenesis, reported in the literature as LD50 values, and published sequences. We performed homology comparison of these sequences using the ExPASy SIM alignment tool for protein sequences and then predicted their 3D structures using the I-TASSER method for protein structure prediction. We retained the best 20 I-TASSER models for the NS1 sequences considered here and compared their structures with that of the X-ray crystallographic structure of the NS1 protein in the A/blue-winged teal/MN/993/1980 (H6N6). The average RMS between this experimental structure and the best 20 I-TASSER models was used as a measure of structural similarity between the 3D structures among the proteins. RESULTS/ANTICIPATED RESULTS: The sequence homology shows modest correlation between sequence and pathogenicity. Linear correlations with R values as large as 0.6 where observed for the full sequence homology and the homology of the RBD domains of the proteins. The correlations with the other protein domains were significant lower. We did not found overall correlation between the 3D structures and pathogenesis of all the variants considered here, but the initial results suggest that correlations do exists for different subgroups of viruses. In future work we will use advanced data mining methods to better understand clustering and correlation between structure and pathogenesis. DISCUSSION/SIGNIFICANCE OF IMPACT: The results presented in this poster demonstrate, as proof of concept, the use of in silico approaches to determine pathogenesis of viruses with substantial impact on human health. The ability of computationally predicting pathogenesis of rapidly mutating viruses can be an effective way to accelerate the development prevention strategies because computational methods are relatively inexpensive and much more scalable than in vivo approaches.

